# Factors influencing resilience and burnout among resident physicians - a National Survey

**DOI:** 10.1186/s12909-021-02950-y

**Published:** 2021-09-29

**Authors:** Cristina Nituica, Oana Alina Bota, John Blebea, Chin-I Cheng, Gus J. Slotman

**Affiliations:** 1 Department of Surgery, College of Medicine, Central Michigan University, 912 S Washington Ave, Suite 1, Saginaw, MI 48601 USA; 2 Department of Psychology, Education and Teacher Training, Faculty of Psychology and Education Sciences, Transylvania University, 56 Nicolae Balcescu, 500019 Brasov, BV Romania; 3 Department of Statistics, Actuarial, and Data Sciences, Central Michigan University, 1200 S. Franklin St., Mount Pleasant, Saginaw, MI 48859 USA; 4 Department of Surgery, Inspira Medical Center Vineland, Inspira Medical Group Surgical Oncology, 1505 West Sherman Avenue, Suite B, Vineland, NJ 08360 USA

**Keywords:** Resident physician, Resilience, Burnout, Survey, National

## Abstract

**Background:**

Residency training exposes young physicians to a challenging and high-stress environment, making them vulnerable to burnout. Burnout syndrome not only compromises the health and wellness of resident physicians but has also been linked to prescription errors, reduction in the quality of medical care, and decreased professionalism. This study explored burnout and factors influencing resilience among U.S. resident physicians.

**Methods:**

A cross-sectional study was conducted through an online survey, which was distributed to all accredited residency programs by Accreditation Council of Graduate Medical Education (ACGME). The survey included the Connor-Davidson Resilience Scale (CD-RISC 25), Abbreviated Maslach Burnout Inventory, and socio-demographic characteristics questions. The association between burnout, resilience, and socio-demographic characteristics were examined.

**Results:**

The 682 respondents had a mean CD-RISC score of 72.41 (Standard Deviation = 12.1), which was equivalent to the bottom 25th percentile of the general population. Males and upper-level trainees were more resilient than females and junior residents. No significant differences in resilience were found associated with age, race, marital status, or training program type. Resilience positively correlated with personal achievement, family, and institutional support (*p* <  0.001) and negatively associated with emotional exhaustion and depersonalization (*p* <   0.001).

**Conclusions:**

High resilience, family, and institutional support were associated with a lower risk of burnout, supporting the need for developing a resilience training program to promote a lifetime of mental wellness for future physicians.

## Background

Post-graduate medical residency training, along with continuing changes in modern healthcare, not to mention the Covid-19 coronavirus pandemic, creates a stressful environment and increased risk of burnout. Burnout is defined as a state of mental exhaustion, depersonalization with a decreased sense of personal achievement and is considered a consequence of high levels of stress combined with very ambitious goals [1]. Evidence during the past decade has documented an almost 2-fold increased level of burnout among healthcare providers in comparison to the general working population with more than half of all physicians reporting at least one symptom of burnout [2]. There is a similar prevalence of burnout among resident physicians in general and among medical and surgical residents [3, 4]. Burnout negatively affects many aspects of physicians’ personal and professional lives. Studies have shown that burnout negatively affects the ability to provide quality medical care to patients, including effective communication, demonstration of empathy and establishing therapeutic relationships with patients [5–7]. On a personal level, burnout significantly diminishes personal wellbeing and may even lead to suicide [8–11].

As a response to this concerning situation among residents in training, resilience is receiving more attention because of its potential to positively influence health and wellbeing and counter the negative effects of burnout [2, 12]. Resilience is recognized as an indicator of psychological maturity [13, 14] and can help residents to cope with the stress inherent in training and their subsequent lives as physicians. Resilient individuals deal more effectively with adversity and the challenges of high workload and high expectations which are characteristics of the medical profession [15–18]. Improving resilience, therefore, can be expected to decrease the development and negative sequel of burnout.

We wished to examine burnout and resilience among U.S. resident physicians in the United States by quantifying the degree of burnout and resilience as well as identifying the demographic and work-related characteristics that are predictive of burnout.

## Methods

A cross-sectional study using an online survey was conducted from November 2018 to January 2019. An email invitation to participate in the survey was sent to all residency training program directors and/or program coordinators listed online by Fellowship and Residency Electronic Interactive Database (FREIDA™) in the United States requesting that they forward the survey link to their residents. The email also included a cover letter to the residents asking for their voluntary participation, explaining the confidentiality of results, and providing a hyperlink to the survey. The respondents completed a baseline questionnaire online that included general demographic information, the Abbreviated Maslach Burnout Inventory (AMBI), the Connor-Davidson Resilience Scale (CD-RISC), questions on compliance with ACGME 80 h duty restrictions, and institutional and family support. The AMBI [19] is an introspective and validated psychological inventory consisting of 9-items pertaining to occupational burnout and incorporates three dimensions: emotional exhaustion (EE), depersonalization (DP), and personal achievement (PA). All AMBI items are scored using a 7-level frequency scale from “never” (0) to “daily” (6). A high score on EE and DP associated with a low score on PA indicates a high level of burnout. The 25-item version of CD-RISC was used to measure resilience [20]. Respondents indicated their level of agreement using a 5-point Likert scale from “strongly disagree” (0) to “strongly agree” (4). The total score was calculated by adding all responses and thus ranges from 0 to 100, with higher scores reflecting greater resilience. The response for family support and compliance with 80 h restriction were using 5-point Likert scale from “never” (1) to “always” (5). The 5-point Likert scale assigned for responses on questions related to job satisfaction including “considering all of this I like my job”, “there is a positive morale at work”, “this hospital is a good place to work”, “I am proud to work at this hospital” and “during my residency I feel like being part of a large family” was from “strongly disagree” (1) to “strongly agree” (5). The response on “number of hours of sleep” used 4-point Likert scale from “4 or fewer hours (4)” to 9 or more hours (1)”. The response on “how comfortable do you feel making autonomous decision in care for the patient” was in 5-point Likert scale from “Not at all comfortable” (1) to “extremely comfortable” (5). The 5-point Likert scale assigned for responses on “how satisfied are you with faculty involvement in your education?” was from “very dissatisfied” (1) to “very satisfied” (5). The response on “the level of supervision during your current year of training” is using 5-point Likert scale from “no supervision” (1) to “direct supervision” (5). We chose a margin of error of 5% and a confidence level of 95% to assess the response rate as adequate with a calculated minimal sample size of 383. The population size was estimated using Association of American Medical Colleges (AAMC) 2019 residency report.

The study was approved by our local Institutional Review Board and the anonymity of the respondents was fully protected with no personal nor program identifiers being collected. Statistical analysis was performed using the SPSS statistical software [IBM Corp, Armonk, NY]. Proportions and frequencies were calculated for categorical variables while means and standard deviation were computed for continuous variables. Comparisons of mean CD-RISC on different groups in gender, age, ethnicity, and relationship in Table 1 were made using one-way ANOVA, respectively. The correlations between CD-RISC and factors of interest were examined by Pearson’s correlation coefficient in Table 3. Multiple linear regression modeled the association between demographic variables and CD-RISC, personnel achievement, emotional exhaustion and depersonalized, respectively. The results were summarized in Table 4. The model assumptions for one-way ANOVA and multiple linear regression were examined and satisfied. Statistical significance was set at *P* <  0.05.

**Table 1:** Demographic characteristics of survey respondents

Variable		n	%	CD-RISC^**a**^ (Mean + SD)	***p***-value
Gender	Female	383	56	71 + 12	0.014
	Male	299	44	74 + 13	
Age (years)	Younger than 35	601	88	72 + 12	0.093
	35 or older	81	12	75 + 13	
Ethnicity	Caucasians	458	67	73 + 12	0.107
	Asian / Pacific Islander	113	17	71 + 13	
	Hispanic	47	7	75 + 12	
	Multiple ethnicity / Other	36	5	69 + 11	
	African American	27	4	74 + 9	
	American Indian or Alaskan Native	1	< 1		
Relationship	Married/ Partnership	452	66	73 + 12	0.560
	Single, never married	208	31	71 + 12	
	Separated/ Divorced/ Widow	22	3	73 + 10	
Training Level	PGY 1	167	25	72 + 12	0.037
	PGY 2	178	26	71 + 12	
	PGY 3	174	26	72 + 12	
	PGY 4	107	16	74 + 12	
	PGY 5	34	5	74 + 9	
	PGY 6	8	1	84 + 5	
	PGY 7	5	< 1	78 + 10	
	PGY 8	9	1	78 + 19	
Type of Program	University Hospital	419	61	73 + 12	0.132
	Community Hospital	230	34	71 + 13	
	Other	33	5	72 + 11	
Geographic Location	Territory (PR)	2	< 1	93 + 0	0.057^†^
	West	84	12	72 + 13	
	South	188	28	74 + 12	
	Mid-West	204	30	72 + 12	
	North-East	204	30	71 + 12	

^a^*CD-RISC* Connor-Davidson Resilience Scale

^†^excluded Territory (PR)

## Results

There was a total of 848 survey respondents. Of these respondents, 682 (81%) completed all the questions and were thus used for further data analysis. This response rate surpassed our calculated minimal sample size requirement of 383. The demographic details about the participants are presented in Table 1.

**Fig. 1:**
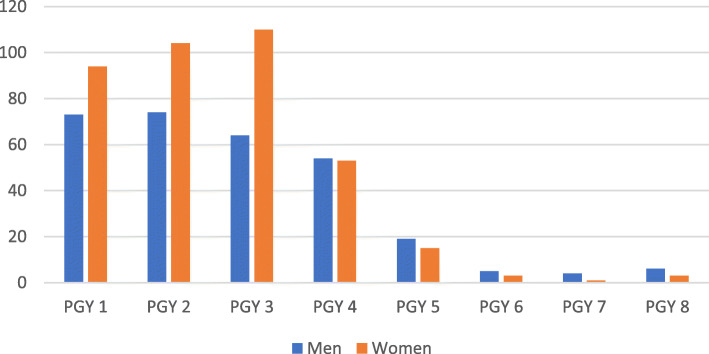
Gender distribution across post graduate year (PGY) training levels. Males labeled in blue, females labeled in orange. PGY1 = residents in first year of postgraduate training, PGY2 = residents in the second year of postgraduate training, PGY3 = residents in the third year of postgraduate training, PGY4 = residents in the fourth year of postgraduate training, PGY5 = residents in the fifth year of postgraduate training, PGY6 = residents in the sixth year of postgraduate training, PGY7 = residents in the seventh year of postgraduate training, PGY8 = residents in the eighth year of postgraduate training

The responders had almost equal gender distribution female (*N* = 383, 56%) as compared to male (*N* = 299, 44%). The majority (*N* = 601, 88%) were in 25–34 years of age, Caucasians (*N* = 458, 67%), and married or in a long-term partnership (*N* = 452, 66%). Gender distribution among training level is depicted in Fig. 1 and reflects the increasing number of graduating medical students, and subsequently residents, being female.

Table 2 describes the specialty distribution of the survey respondents. Three quarters, (*N* = 509, 75%) were in medical specialties while the remainder were surgical residents. A comparison of all residents, reflected in the 2019 AAMC resident distribution by specialty data, indicates that the respondents on the survey were broadly representative of all residents in the U.S.

**Table 2:** Specialty distribution of respondents versus all residents in U.S

Specialty	Survey Respondents					2019 AAMC Data				
	Male	%	Female	%	Total	Male	%	Female	%	Total
Anesthesiology	22	56	17	44	39	4023	66	2034	34	6057
Child Neurology	2	22	7	78	9	123	32	266	68	389
Dermatology	3	50	3	50	6	562	39	877	61	1439
Diagnostic Radiology-Nuclear Medicine	4	50	4	50	8	4	67	2	33	6
Emergency Medicine	31	61	20	39	51	4941	65	2720	36	7661
Emergency Medicine-Family Medicine	2	100	0	0	2	18	50	18	50	36
Family Medicine	17	32	37	69	54	5735	46	6663	54	12,398
Family Medicine-Preventive Medicine	1	100	0	0	1	10	50	10	50	20
Internal Medicine	21	46	25	54	46	15,389	58	11,284	42	26,673
Internal Medicine-Emergency Medicine	1	50	1	50	2	85	64	47	36	132
Internal Medicine-Medical Genetics	0	0	1	100	1	4	80	1	20	5
Internal Medicine-Pediatrics	4	29	10	71	14	606	41	874	59	1480
Internal Medicine-Preventive Medicine	1	100	0	0	1	14	48	15	52	29
Internal Medicine-Psychiatry	2	100	0	0	2	56	53	49	47	105
Interventional Radiology-Integrated	2	40	3	60	5	172	80	43	20	215
Medical Genetics and Genomics	0	0	1	100	1	22	34	43	66	65
Neurology	9	69	4	31	13	1516	55	1266	46	2782
Neurological Surgery	9	82	2	18	11	1218	83	259	18	1477
Obstetrics and Gynecology	7	12	54	89	61	886	17	4495	84	5381
Ophthalmology	8	47	9	53	17	794	60	538	40	1332
Orthopedic Surgery	18	75	6	25	24	3353	85	610	15	3963
Otolaryngology-Head and Neck Surgery	2	40	3	60	5	1025	64	581	36	1606
Pathology -Anatomic and Clinical	4	31	9	69	13	1125	50	1120	50	2245
Pediatrics	19	25	57	75	76	2461	28	6419	72	8880
Pediatrics-Anesthesiology	1	100	0	0	1	13	34	25	66	38
Pediatrics-Physical Medicine and Rehabilitation	0	0	2	100	2	2	17	10	83	12
Pediatrics-Psychiatry-Child and Adolescent Psychiatry	0	0	3	100	3	22	24	71	76	93
Physical Medicine and Rehabilitation	8	57	6	43	14	843	63	503	37	1346
Plastic Surgery	2	100	0	0	2	142	69	63	31	205
Plastic Surgery-Integrated	1	33	2	67	3	524	59	372	42	896
Preventive Medicine	4	50	4	50	8	142	49	146	51	288
Psychiatry	21	35	39	65	60	2934	50	2943	50	5877
Psychiatry-Family Medicine	2	50	2	50	4	18	35	33	65	51
Radiation Oncology	10	67	5	33	15	519	70	225	30	744
Radiology-Diagnostic	12	44	15	56	27	3194	73	1178	27	4372
Surgery - General	21	53	19	48	40	5384	59	3789	41	9173
Thoracic Surgery-Integrated	1	100	0	0	1	158	73	59	27	217
Transitional Year	8	57	6	43	14	798	63	464	36	1262
Urology	14	74	5	26	19	1009	75	342	25	1351
Vascular Surgery-Integrated	5	71	2	0.3	7	212	67	107	,34	319
Total	299	44	383	56	682	60,056	54	50,564	46	110,620

Descriptive statistics for the Connor-Davidson Resilience Scale showed a mean value of 72 with a median of 72 and a mode of 65. There were no significant differences in CD-RISC scores based on age, ethnicity, or marital status (Table 1). However, female residents were significantly less resilient (F = 6.103, *p* = 0.014) when compared to their male counterparts, with a score of 71 and 74, respectively.

No significant differences in resilience were found among participants from academic versus community hospital-based training program (F = 2.031, *p* = 0.132) or geographic regions (F = 2.522, *p* = 0.057). The residents in the upper level of training had significantly higher CD-RISC scores when compared to the junior residents (F = 2.145, *p* = 0.037) with residents from postgraduate years six to eight (PGY 6–8) being the most resilient with CD-RISC = 80.1 (13.4), followed by the residents from postgraduate year four and five (PGY 4–5) with CD-RISC = 74.1(11.3) and postgraduate year one to three (PGY 1–3) with CD-RISC = 71.6 (12.5).

Specialty distribution was also not found to be correlated to with resilience (F = 1.176, *p* = 0.250). However, when comparing the medical and surgical specialties, surgical residents scored higher in resilience than medical residents (F = 7.169, *p* = 0.008; CD-RISC = 74.5 (11.5) versus 71.7 (12.3).

There was a significant and positive correlation between family support and higher resilience (*r* = 0.28, *p* <  0.001; Table 3).

**Table 3:** Associations between factors and resilience (Pearson correlation of CD-RISC) (*n* = 682)

Factors	Factor-resilience relationship	
	***r***	***p-value***
Family support	0.28	<0.001
Considering all of this I like my job	0.50	<0.001
Compliance with 80 h restriction	0.13	< 0.001
Personal achievement	0.48	< 0.001
Emotional exhaustion	−0.48	< 0.001
Depersonalization	− 0.30	< 0.001
Number of hours of sleep	−0.01	0.720

Residents with strong family support (always, usually) scored higher than the residents with sporadic or inexistent family support (sometimes, rarely, never). Job satisfaction and residency program support was assessed through five questions and was also found to correlate positively with resilience. There is a positive correlation with the self-affirmation “Considering everything I like my job “(*r*= 0.50, *p*< 0.001), “There is a positive morale at work“ (*r*= 0.39, *p*<0.001), “This hospital is a good place to work“ (*r*=0.36, *p*<0.001), “I am proud to work at this hospital“ (*r*= 0.37, *p*<0.001)”, and “During my residency I feel like being part of a large family” (*r* = 0.33, *p* < 0.001). No correlation was found between the resilience index and the number of hours of sleep (*r* = − 0.01, *p* = 0.720), however the compliance with the 80-h restriction was a small but significant correlate (*r* = 0.13, *p* <  0.001).

Multiple linear regression showed five significant factors associated with higher resilience (Table 4): family support, geographic location, surgical specialties, autonomy, and agreeing to the question “Considering everything, I like my job“.

**Table 4:** Multiple linear regression analysis of variables relating resilience, personal achievement, emotional exhaustion and depersonalization

Source	CD-RISC		Personal Achievement		Emotional Exhaustion		Depersonalization	
	Beta	***p***-value	Beta	***p***-value	Beta	***p***-value	Beta	***p***-value
CD-RISC			0.03	<0.001	−0.02	< 0.001	−0.01	0.017
Family support	1.85	<0.001	−0.04	0.282	0.03	0.405	< −0.01	0.914
Autonomy	3.47	<0.001	0.16	<0.001	0.02	0.691	0.01	0.837
Considering everything I like my job	4.66	<0.001	0.22	<0.001	−0.29	<0.001	−0.16	0.003
Surgical Specialties								
Non-Surgical	−3.31	<0.001	0.04	0.63	0.03	0.652	−0.07	0.427
Surgical	Reference							
Geography		0.007		0.838		0.807		0.104
Mid-West	−0.50	0.689	−0.01	0.953	0.04	0.669	0.01	0.928
North-East	−0.72	0.565	0.04	0.698	−0.03	0.790	−0.17	0.153
South	2.43	0.055	−0.04	0.726	−0.02	0.823	−0.17	0.147
West	Reference							
I am proud to work at this hospital	0.94	0.125	0.06	0.305	−0.1	0.041	−0.13	0.022
There is a positive morale at work	0.95	0.107	<0.01	0.972	−0.15	0.001	−0.01	0.856
Gender								
Female	−1.16	0.127	0.12	0.082	0.09	0.124	−0.43	<0.001
Male	Reference							
Marital Status		0.331		0.102		0.073		0.488
Married	−1.21	0.152	−0.14	0.066	−0.15	0.026	−0.05	0.531
Separated	−1.69	0.446	0.11	0.579	−0.01	0.957	−0.24	0.250
Single	Reference							
Type of program		0.332		0.751		0.887		0.543
Community	−0.62	0.458	−0.05	0.540	0.02	0.76	−0.09	0.269
Other	−2.41	0.168	0.05	0.726	0.06	0.673	−0.02	0.899
University	Reference							
Age								
35 and older	1.51	0.201	0.15	0.156	−0.05	0.575	−0.23	0.034
Younger than 35	Reference							
Race		0.396		0.681		0.010		0.006
African American	1.27	0.515	−0.05	0.762	−0.05	0.747	−0.55	0.002
American Indian	2.80	0.773	0.24	0.776	0.54	0.49	−0.40	0.662
Asian	−0.75	0.469	−0.13	0.164	−0.27	0.001	−0.09	0.379
Hispanic	2.04	0.174	−0.14	0.296	−0.14	0.249	−0.35	0.012
Other	−2.29	0.173	−0.13	0.396	0.19	0.153	0.14	0.368
Caucasians	Reference							
Satisfaction with faculty	0.18	0.723	0.02	0.718	−0.04	0.320	−0.02	0.660
Supervision	−0.80	0.109	0.04	0.404	0.04	0.312	0.04	0.385
This hospital is a good place to work	0.41	0.507	<0.01	0.982	−0.05	0.281	−0.12	0.039
Compliance with 80 h rule	0.44	0.381	−0.04	0.368	−0.06	0.132	0.03	0.568
During my residency I feel being part of a big family	−0.01	0.834	0.04	0.303	0.02	0.591	0.03	0.550

The average CD-RISC score for residents increased by 1.85 points for every one-point increase in Likert scale in family support. The average CD-RISC score for residents increased by 3.47 points for every one-point increase in Likert scale in comfortable being autonomous in making medical decisions. For every one-point increase in Likert scale regarding the question” Considering everything, I like my job”, the average CD-RISC score increases by 4.66 points. Overall, 64% of the respondents were found to have at least one element of burnout with predominance on emotional exhaustion (58%). Resilience positively correlates with the sense of personal achievement (*r* = 0.484, *p* < 0.001) and negatively with emotional exhaustion (*r* = − 0.477, *p* <  0.001) and depersonalization (*r* = − 0.305, *p* < 0.001).

Each element of burnout was examined using multiple linear regression. Personal achievement was positively corelated with autonomy, “Considering everything, I like my job”, and having higher resilience score. Emotional exhaustion had five significant factors: race, disagreeing with the questions “Considering everything, I like my job,” “There is a positive morale at work,” “I am proud to work at this hospital,” and a low CD-RISC. The emotional burnout for White/Caucasians residents was higher than that for Asian/Pacific islander residents (*p* < 0.001). Although not significant in the multiple linear regression analysis, the emotional exhaustion for residents that were “single/never married” was higher than that for “married/in a partnership” residents (*p* = 0.026).

We found six significant factors in the multiple linear regression analysis influencing depersonalization: resident under age 35 years (*p* = 0.034), male gender (*p* <  0.001), race (*p* = 0.006), lower CD-RISC (*p* = 0.017), disagreeing with “Considering everything, I like my job” (*p* = 0.003), and “This hospital is a good place to work” (*p* = 0.039). Caucasians residents reported higher depersonalization when compared to Hispanics (*p* = 0.012) and African Americans residents (*p* = 0.002).

## Discussion

This study was conducted based on the premise that resident physicians must navigate a complex, contradictory, and stressful environment which makes them vulnerable to burnout. There is ample literature supporting the concept that resilience is inversely correlated with burnout [5, 21, 22]. In addition, there is genuine concern among academic faculty that there is decreasing resilience among graduate and post-graduate students in the United States that extends to resident physicians. By extension, residents with higher levels of resilience would be expected to better cope and adapt to the stresses of residency. Our study examined to what degree this expectation is correct.

In the original Connor and Davidson 2003 study, mean CD-RISC scores for the U.S. general population was 81, with quartile percentile distribution for Q1, Q2, Q3, and Q4 being 0–73, 74–82, 83–90, 91–100 [20]. In comparison, score means for primary care patients and psychiatric outpatients were 72 and 68, respectively. In this context, the resident physician participants from this study had a median of 72, placing them in the lowest 25% of the general population and at a similar level to older primary care patients. Our results are also similar to a prior study that examined resilience in interns [21].

Our results did not demonstrate any difference in CD-RISC resilience scores based on age, marital status, or ethnicity. This is consistent with the findings summarized by Davidson [23].

and in the general U.S. population [20]. There were, however, gender differences. We found that male resident physicians were more resilient than females (CD-RISC score of 74 vs 71). Such gender differences vary among different populations and is inconsistent. Connor found no gender differences in the general population [20] but among medical students, male had higher resilience scores than female in both Canadian [24] and U.S. medical students [25]. Perhaps reflecting a selection bias, female Air Force recruits were more resilient than male [26].

No significant resiliency differences were found among participants from different types of training programs (academic vs. non-academic), specialty or geographic regions. No prior published literature has focused on these characteristics. Although age was not a significant factor for resilience, as also noted in other groups [20, 27] the level of training was. Upper-level residents were more resilient than junior residents. PGY 1–3 had CD-RISC scores corresponding to the 25th percentile of the U.S. population while PGY 4–5 improved to the level of the 50th percentile and those in PGY 6–8 were close to 75th percentile. These findings suggest that resilience does not increase with age but rather is enhanced by experience and speaks of the positive effect of the residency training environment.

Family support and friends had a significant and positive effect on increasing resilience, as also seen in other populations [7, 28, 29]. In addition, resilience positively correlated with personal achievement (*p* < 0.001) and negatively with emotional exhaustion and depersonalization (*p* <  0.001). Similar evidence is found in the literature [25, 30–33] and suggests that interventions addressing these areas can improve resilience during residency and thus prevent burnout in our trainees.

Almost two thirds of the survey respondents had at least one element of burnout with a predominance reporting emotional exhaustion. Previously, others had reported burnout from 40 to 75% among U.S. residents [25] comparable with global burnout prevalence of over 50% in other populations [25]. We further found that being single was associated with emotional exhaustion and Caucasians experienced more emotional exhaustion and depersonalization than other ethnic groups.

Our study has several limitations. Although the number of respondents was almost double the required minimum sample size, the overall response rate was low. This is explained by program contact information that was not 100% accurate so that some of the survey requests did not reach their destination. Without direct contact information for the individual residents, we relied on the program directors or coordinators to forward the survey to their trainees, which may not have occurred in many cases due to the large number of survey requests being sent out to programs. The response rate from various groups representing ethnicity, geographic location, and specialties is challenging to calculate but appears to reflect the national AAMC data. Future studies, such as the ACGME directed survey, could include more extensive resilience and burnout inventory scales. Nonetheless, our results are consistent with other studies and suggest foci for attention to increase resilience and decrease burnout in our resident physicians.

## Conclusions

This study brings compelling evidence that resilience development should be done not only by teaching individuals to be resilient but also by developing the infrastructure and institutional protective support system against burnout in healthcare providers.

## Data Availability

The datasets used and/or analyzed during the current study are not immediate available due to technical support availability but it is freely obtainable from the corresponding author on request, given reasonable time to obtain the necessary technical support.

## Abbreviations

ACGME: Accreditation Council of Graduate Medical Education
AMBI: Abbreviated Maslach Burnout Inventory
ANOVA: Analysis of Variance
CD-RISC 25: Connor-Davidson Resilience Scale
DP: Depersonalization
EE: Emotional Exhaustion
FREIDA™: Fellowship and Residency Electronic Interactive Database
IBM Corp: International Business Machines Corporation
PA: Personal Achievement
PGY 6–8: Postgraduate Year six to eight
PGY 4–5: Postgraduate Year four and five
PGY 1–3: Postgraduate Year one to three
SPSS: Statistical Product and Service Solution
